# Preface to special topic on new era of zeolite science

**DOI:** 10.1093/nsr/nwac157

**Published:** 2022-08-17

**Authors:** Jihong Yu, Dongyuan Zhao

**Affiliations:** State Key Laboratory of Inorganic Synthesis and Preparative Chemistry, College of Chemistry, International Center of Future Science, Jilin University, China; Department of Chemistry, State Key Laboratory of Molecular Engineering of Polymers, Shanghai Key Laboratory of Molecular Catalysis and Innovative Materials, Laboratory of Advanced Materials, and Collaborative Innovation Center of Chemistry for Energy Materials (iChEM), Fudan University, China

Zeolites are crystalline microporous solids whose frameworks are composed of corner-sharing tetrahedral TO_4_ units (T = Si, Al, P, etc.), forming periodic one-dimensional (1D) to three-dimensional (3D) channels with an aperture size of typically <2 nm. Because of their unique pore structures, large specific surface area, strong acid sites, high thermal/hydrothermal stability and molecular-level shape selectivity, zeolites are widely used as catalysts, adsorbents and ion exchangers in the fields of oil refining, petrochemical industry, coal chemical industry and daily chemical industry.

Almost 60 years have passed since the first industrial application of Y zeolite in oil cracking, which caused the ‘technical revolution in the refinery industry’. To date, the Structure Committee of the International Zeolite Association has certified over 250 types of zeolite structures, of which nearly 20 have achieved industrial application, leading to a series of milestone technological revolutions in energy, chemical and environmental fields, as well as other fields, in the past few decades. For example, titanium silicalite (TS-1) zeolites can catalyze propylene epoxidation directly to propylene oxide under mild conditions, which promotes the generation and development of green chemistry and atomic economy processes and is known as a ‘green catalytic milestone’; zeolite Cu-SSZ-13 can efficiently catalyze selective reduction of NO_x_ in heavy duty diesel vehicle exhaust, which can significantly reduce exhaust emissions; highly efficient catalytic conversion of methanol to ethylene and propylene by zeolite SAPO-34 makes coal an important raw material for the production of bulk basic organic chemicals; the lithium-ion-exchanged low-silica X zeolite has greatly improved oxygen production efficiency via pressure swing adsorption (PSA), which provides support for low-cost oxygen use in many arenas, such as the chemical industry and steel, cement and medical treatment fields; a composite of partially reduced oxide (ZnCrOx) and mesoporous SAPO-34 zeolite (MSAPO) broke the Anderson-Schulz-Flory (ASF) limit in a direct synthesis gas (syngas) conversion to light olefins (C_2_^=^ –C_4_^=^) via Fischer-Tropsch synthesis (FTS); and very recently, ultrathin membranes of Li-exchanged X zeolites were used as chemically stable solid electrolytes, which gave the resultant solid-state Li–air battery a greater cycle life than batteries based on conventional solid electrolytes and organic electrolytes under the same conditions.

In recent years, with the progress of synthetic chemistry, the development of characterization technologies and theoretical calculations, and interdisciplinary integration, global zeolite research has experienced a new upsurge. Breakthrough progress has been made in the discovery of new zeolites, zeolite structure characterization, theoretical simulations, efficient catalysis and adsorptive separation. Progress has also been made with regard to new applications in energy storage, optoelectronic devices, biomedicine and biomass conversion. To highlight recent developments and crucial issues for future research in zeolite science, we have organized this special-topic issue, ‘New Era of Zeolite Science’.

This special topic includes one perspective, four research articles, five reviews and one interview. In the perspective, Mintova *et al*. show the exploration, explanation and exploitation of hydroxyls in zeolites.

In the research articles, Corma *et al.* illustrate the enzymatic and chemo-enzymatic strategies of producing highly valuable chiral amines from biomass with ω-transaminases on 2D zeolites; Yu *et al.* unravel the templated-regulated distribution of isolated SiO_4_ tetrahedra in silicoaluminophosphate zeolites with high-throughput computations; Liu *et al.* present the multiscale dynamical cross-talk in zeolite-catalyzed methanol and dimethyl ether conversions; and Bao *et al.* show the dynamic confinement of SAPO-17 cages on the selectivity control of syngas conversion.

In the reviews, Weckhuysen *et al.* review emerging analytical methods of characterizing zeolite-based materials; Xiao *et al.* summarize the targeted synthesis of zeolites from calculated interaction between zeolite structure and organic template; Deng *et al.* give an overview of recent advances in solid-state NMR of zeolite catalysts; Wu *et al.* present new progress in zeolite synthesis and catalysis; and Yu *et al.* review low-energy adsorptive separation by zeolites.

In the interview, Prof. Ruren Xu, an early leader within Chinese, Asian and worldwide zeolite communities, the founder of the inorganic synthesis discipline in China and the first proposer of the scientific discipline of modern inorganic synthetic chemistry in the world, highlights frontiers in zeolites towards establishing a new discipline of condensed matter chemistry.

We thank all the authors and editorial staff for their efforts in making this theme possible. Special thanks goes to Prof. Ruren Xu and Prof. Wenfu Yan for the interview. Zeolite is an old material, however, its application has been extended to many sustainable processes, which promises a brilliant new future.

